# PLACE OF DEATH AMONG MEDICARE BENEFICIARIES: VARIATIONS BY DEMENTIA DIAGNOSIS AND RACE/ETHNICITY

**DOI:** 10.1093/geroni/igac059.1399

**Published:** 2022-12-20

**Authors:** Olga Jarrín, Haiqun Lin, Bei Wu, Anum Zafar, Maria Lopez, Paul Duberstein

**Affiliations:** Rutgers, The State University of New Jersey, New Brunswick, New Jersey, United States; Rutgers University, Newark, New Jersey, United States; New York University, New York, New York, United States; Rutgers, The State University of New Jersey, New Brunswick, New Jersey, United States; Rutgers, The State University of New Jersey, New Brunswick, New Jersey, United States; Rutgers School of Public Health, Piscataway, New Jersey, United States

## Abstract

Death at home is preferred by most people and has become a key indicator of the end-of-life care quality; however cultural attitudes about death and dying may vary by race and ethnicity. This study describes racial and ethnic variation in place of death among Medicare beneficiaries aged 50 years and older who died in 2018 with a dementia diagnosis (n=902,208) and without a dementia diagnosis (n=1,096,074). Among those with dementia, 60% died at home, with the highest percentage for non-Hispanic white (62%) beneficiaries, followed by Black/African American (55%), Hispanic (61%), Asian American/Pacific Islander (58%), and American Indian/Alaska Native (58%) beneficiaries. Among those without dementia 61% died at home, with the highest percentage for non-Hispanic white (60%) beneficiaries, followed by Black (57%), Hispanic (65%), Asian American (65%), and American Indian (60%) beneficiaries. Among those who died at home, about half without dementia received hospice care compared to the majority with dementia.

